# Characteristics of Young Women Who Gave Birth in the US-Mexico Border Region, 2005: The Brownsville-Matamoros Sister City Project for Women’s Health

**Published:** 2008-09-15

**Authors:** Jill A. McDonald, Francisco Gerardo Galván González, Gita G Mirchandani, Brian C Castrucci, Ginger L Gossman, Kayan L Lewis, Mauro Ruiz, Alonso Echegollen Guzmán

**Affiliations:** Centers for Disease Control and Prevention, National Center for Chronic Disease Prevention and Health Promotion, Division of Reproductive Health; Instituto Mexicano del Seguro Social, Coordinación Delegacional de Salud Reproductiva, Ciudad Victoria, Tamaulipas, Mexico; Texas Department of State Health Services, Division of Family and Community Health Services, Office of Title V, Austin, Texas; Texas Department of State Health Services, Division of Family and Community Health Services, Office of Title V, Austin, Texas; Texas Department of State Health Services, Division of Family and Community Health Services, Office of Title V, Austin, Texas; Texas Department of State Health Services, Division of Family and Community Health Services, Office of Title V, Austin, Texas; Texas Department of State Health Services, Region 11, Harlingen, Texas; Instituto Mexicano del Seguro Social, Coordinación Delegacional de Investigación en Salud, Ciudad Victoria, Tamaulipas, Mexico.

## Abstract

**Introduction:**

Childbearing during adolescence and young adulthood is associated with adverse effects on health and quality of life. Lowering birth rates among young women is a binational priority in the US-Mexico border region, yet baseline information about birth rates and pregnancy risk is lacking. Increased understanding of the characteristics of young women who give birth in the region will help target high-risk groups for sexual and reproductive health services.

**Methods:**

We examined data on reproductive health characteristics collected in hospitals from 456 women aged 24 years or younger who gave birth from August 21 through November 9, 2005, in Matamoros, Tamaulipas, Mexico, and Cameron County, Texas. We calculated weighted percentages and 95% confidence intervals (CIs) for each characteristic and adjusted odds ratios (AORs) for Matamoros and Cameron County women by using multiple logistic regression techniques.

**Results:**

Numbers of births per 1,000 women aged 15 to 19 years and 20 to 24 years were similar in the 2 communities (110.6 and 190.2 in Matamoros and 97.5 and 213.1 in Cameron County, respectively). Overall, 38.5% of women experienced cesarean birth. Matamoros women reported fewer prior pregnancies than did Cameron County women and were less likely to receive early prenatal care but more likely to initiate breastfeeding. Few women smoked before pregnancy, but the prevalence of alcohol use in Cameron County was more than double that of Matamoros. In both communities combined, 34.0% of women used contraception at first sexual intercourse.

**Conclusion:**

Despite geographic proximity, similar ethnic origin, and comparable birth outcomes, young Mexican and US women showed different health behavior patterns. Findings suggest possible pregnancy prevention and health promotion interventions.

## Introduction

Childbearing and parenting among adolescents and young adult women is associated with adverse effects on health and quality of life for both mother and infant (1,2). The birth rate for young US women of Hispanic origin is higher than that for young US women overall: in 2006, the birth rate for Hispanic women aged 15 to 19 years was 83 per 1,000, compared with 42 per 1,000 for all US women in the same age group. The birth rate for Hispanic women aged 20 to 24 years was 177 per 1,000, compared with 106 per 1,000 for all US women in this age group (3). Adolescent birth rates are high in US counties on the Mexican border, where Hispanic concentration is high (4,5). In 2004, for example, the birth rate among women aged 15 to 19 years was 62 per 1,000 in Texas and 96 per 1,000 in the border's southernmost county, Cameron County (J. Jackson, MPH, written communication, February 2008).

The birth rate among young Mexican women is also high. Vital statistics data from 2006 indicate a national rate of 74 per 1,000 women aged 15 to 19 years. Similar adolescent birth rates are documented in the border state of Tamaulipas (75 per 1,000), which borders Texas, and in the municipality of Matamoros, Tamaulipas (75 per 1,000), which is directly across the international boundary from Cameron County (Figure). Birth rates among women aged 20 to 24 years in 2006 were 140 per 1,000 in Mexico, 139 per 1,000 in Tamaulipas, and 126 per 1,000 in Matamoros (6,7).

**Figure.**
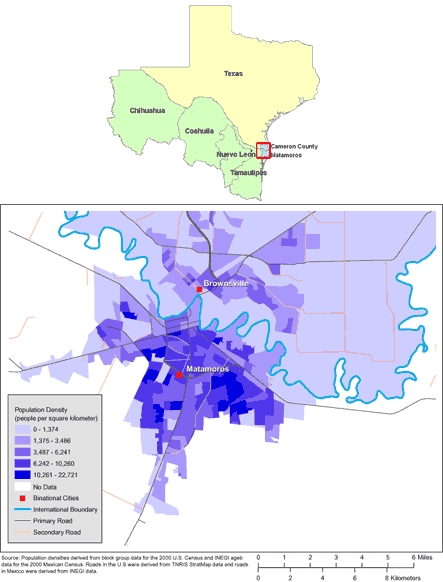
Maps of the US-Mexico Border Region (Top) and of Brownsville, Texas, and Matamoros, Tamaulipas, Mexico (Bottom). (The authors thank Allison Abell Banicki of the Office of Border Health, Texas Department of State Health Services, for creating the map of the Texas-Mexico border states and thank Jean W. Parcher, Sylvia N. Wilson, and the United States Geological Survey [USGS] for providing the map of population density in Brownsville and Matamoros.)

In part because of these statistics, the United States-Mexico Border Health Commission has set objectives for reducing adolescent birth rates on both sides of the border and improving the delivery of prenatal care to women of all ages by 2010 (8). The target is a 20% reduction in births among adolescents in the border region of Mexico and a 33% reduction in the border region of the United States (8).

Unfortunately, little more than the overall rates is known about births among adolescents and other young women in the US-Mexico border region, and the reliability of those rates is unclear, especially in the Mexican states where state governments are actively engaged in campaigns to increase birth registration (9). Such information is critical for planning and evaluating health education and teenage pregnancy prevention programs created for this population. Insufficient family planning resources are available for adolescents and young women in border communities (B.R. Smith, MD, MPH, written communication, March 2008).

To provide the information needed for such programs, the Centers for Disease Control and Prevention (CDC) recently developed the Brownsville-Matamoros Sister City Project for Women's Health (BMSCP), a model for reproductive health risk factor surveillance in border communities, in collaboration with governmental health institutions in Tamaulipas and Texas and other community partners (10). The BMSCP pilot survey, conducted in 2005, covered a range of reproductive and chronic disease indicators, including data on prepregnancy, prenatal, and birth experiences. The survey interviewed a representative sample of women who gave birth in the municipality of Matamoros or in Cameron County, where the city of Brownsville is. We used the BMSCP data to 1) provide another measure of age-specific birth rates among young women in the 2 communities, 2) describe the sociodemographic and reproductive health characteristics of young women who gave birth in each community and overall, and 3) compare findings between the 2 communities.

## Methods

This surveillance pilot project was reviewed for human subjects concerns by CDC and was determined to be "nonresearch" or public health practice. Therefore, institutional review board approval was not required. Data were collected between August 21 and November 9, 2005, through a hospital-based, postpartum survey of women who gave birth in Matamoros or Cameron County (10). Briefly, we used a stratified cluster sampling design to select women for interview who delivered a live infant in either community in a hospital that experienced 100 or more deliveries in 2004. All women who delivered a live infant on selected days were included in the sample. Sampled women were identified through review of hospital delivery logs. Retrospective review of vital statistics data showed that the sampling approach included 95% of the birth population and that more than 97% of women in each community who delivered live infants in study hospitals on sample days were successfully sampled. Among the 999 women sampled, 947 (95%) completed interviews. In the current analysis, we include only BMSCP respondents aged 24 years or younger (n = 456).

We calculated age-specific birth rates within each community for girls and women aged 10 to 19, 15 to 19, and 20 to 24 years, and then for all girls and women aged 10 to 24 years. The number of women aged younger than 15 years was too small for separate analysis. Survey data were weighted to approximate the number of women who gave birth in each community during the 81-day study period. We derived annualized estimates of the number of births in the population in 2005 and corresponding 95% confidence intervals (CIs) by multiplying the weighted population estimate and associated standard error by 4.51 (365 days/81 study period days). We used age- and sex-specific midyear population estimates for July 1, 2005, as denominators for the rates (11,12).

We calculated birth rate estimates from vital statistics and census data as a comparison. For Cameron County, we used provisional 2005 birth counts, the most recent we could obtain (J. Jackson, MPH, written communication, February 2008). For Matamoros, we used 2006 birth data and population estimates because 2005 birth data were not available (6,7).

Weighted frequencies and proportions were calculated for sociodemographic and reproductive health characteristics for all women in Matamoros, Cameron County, and the combined sample. We analyzed 9 sociodemographic variables from the survey: age, ethnicity, place of birth, interview language, marital status, education, employment status, health care coverage before pregnancy, and health care coverage during pregnancy. Ethnicity was based on self-report in Cameron County, whereas all mothers who lived in Mexico were coded as being Hispanic. Interview language was coded as Spanish if the respondent opted for a Spanish-language interview or used Spanish at any point during the interview. Employment status referenced the 3 months before pregnancy; we classified respondents as 1) employed (employed for wages or self-employed), 2) unemployed (out of work), or 3) not in the labor market (ie, homemaker, student, retired, or unable to work). Small numbers prevented analyses of these variables by age group. We tested for sociodemographic differences between the 2 communities using the Pearson χ^2^ test, with a P value of ≤.05 as the cutoff for statistical significance.

We examined 16 reproductive health characteristics, including 6 pregnancy characteristics: gravidity, intention of pregnancy, low birth weight (<2,500 g), preterm birth (<37 weeks' gestation), method of delivery, and breastfeeding initiation. Unintended pregnancy was defined as a pregnancy the respondent said she would have liked to have later or not at all. Women who responded that they began prenatal care within the first 13 weeks of pregnancy were defined as having had early prenatal care. Those responding yes to the question, "During any of your prenatal care visits, did a doctor, nurse, or other health care worker talk with you about birth control methods to use after your pregnancy?" were coded as having received postpartum contraception counseling.

Respondents were asked about risk behavior and knowledge related to contraception, HIV, smoking and drinking, and history of a Papanicolaou (Pap) test. Depending on their unprompted responses to the question, “What can a person do to protect himself or herself from getting HIV/AIDS?” we coded each respondent as knowing both, 1, or neither highly effective method of HIV prevention: 1) using a condom and 2) limiting sex/staying faithful to a single partner. We used an aggregate measure of “high-risk behavior,” defined as experiencing 1 or more of the following during the previous year: 1) intravenous drug use, 2) treatment for a sexually transmitted infection (STI), and 3) more than 2 sex partners. Respondents were not asked to identify which behaviors applied to them but only to respond positively if 1 or more applied. Smoking and alcohol status were obtained from questions that asked how many cigarettes were smoked on an average day and how many drinks were consumed in an average week during the 3 months before pregnancy. We coded women as smokers or drinkers if they reported any level of consumption. Most survey questions were taken or adapted from established surveys (10). Birth weight was obtained from the hospital record, and gestational age was obtained from the birth certificate. All other variables were derived from the survey questionnaire.

We computed unadjusted odds ratios (ORs), 95% CIs, and P values for the associations between place of residence and each reproductive health characteristic. We used multivariate logistic regression techniques to calculate the adjusted odds ratio (AOR), 95% CI, and P value for each characteristic, adjusted for an a priori set of sociodemographic variables (13), including age (continuous), ethnicity (2-category), marital status (3-category), education (3-category), and health care coverage before pregnancy (2-category). For characteristics with more than 2 outcome categories, we used multinomial logistic regression (14). We conducted all analyses with Stata software, version 9 (StataCorp LP, College Station, Texas), taking survey weights and the complex survey design variables into account.

## Results

Among the 456 women included in this study, 248 reported living in Mexico and were presumed to be Matamoros residents, and 207 reported living in the United States and were presumed to be residents of Cameron County. One other respondent who delivered in Cameron County had a missing response and was classified as a US resident. Among the women who resided in Mexico, 4% (11/248) delivered in a Cameron County hospital. Only 1 of 208 Cameron County residents delivered in a Matamoros hospital. 

Annual birth rates per 1,000 Matamoros women aged 15 to 19 years and Cameron County women aged 15-19 years were similar (Table 1). Annual birth rates among women aged 20 to 24 years were approximately twice those in the younger age group for both communities. In Matamoros, the birth rate in each age group as determined by vital statistics fell below the 95% CI of the birth rate derived from the study.

One-third of women in the study were younger than 20 years (Table 2). Ninety-four percent were Hispanic; Mexico was the birthplace of 99.5% of the Matamoros women and 40.4% of the Cameron County women. Matamoros mothers were less educated but were more likely to be married/living together and to have health care coverage before pregnancy. Overall, approximately two-thirds of women had health care coverage during their pregnancy.

Fewer Matamoros women reported prior pregnancies (Table 3). In each community, fewer than half of the pregnancies were intended. Although Matamoros mothers had less frequent low-birth-weight and preterm births, these differences were not statistically significant. The proportion of cesarean births reached almost 40% in each community. Matamoros mothers were 4 times as likely as Cameron County mothers to have initiated breastfeeding by the time of interview.

Virtually all women had some prenatal care (data not shown), but more Cameron County women (69.9%) than Matamoros women (57.9%) had first-trimester prenatal care. Counseling for postpartum contraception was more frequently a part of prenatal care for Matamoros women (69.4%) than for Cameron County women (58.8%).

The mean age at first sexual intercourse among the 440 women who provided a response was 16.9 years in Mexico and 16.5 years in the United States (*P* = .09). Among women aged 20 years or younger, the mean ages were 15.8 years in Matamoros and 15.6 years in Cameron County (*P* = .22) (data not shown).

Women residing in Matamoros were less likely than women residing in Cameron County to use contraception at first sexual intercourse, but the association was attenuated in the adjusted analysis. More Matamoros women than Cameron County women used an intrauterine device (IUD) as their first method of contraception. Barrier methods were the most common choice in each community.

Use of alcohol before pregnancy was more prevalent among Cameron County women. Only 6% of women in each community reported using intravenous drugs, having been treated for an STI, or having had more than 2 sexual partners in the past year. Fewer women in Mexico reported ever receiving a Pap test.

## Discussion

One purpose of this study was to compare rates calculated via this sample survey with rates from vital statistics as evidence of the validity of published birth rates in both communities. Results suggest that vital statistics in Matamoros may underestimate the true birth rates. In Cameron County, estimates from the survey and vital statistics were more compatible. This may be in part because our survey estimates included births to Matamoros residents that occurred in Cameron County. Those infants may not have received Tamaulipas birth certificates, and Texas does not routinely share birth certificates of infants of nonresident mothers with Tamaulipas. This factor would not have affected Cameron County rates because so few Cameron County residents gave birth in Mexico, and births to Mexican residents in Cameron County are not counted in the vital statistics rates.

Although birth rates generated by this surveillance system were comparable in the 2 communities, more Cameron County women than Matamoros women reported a previous pregnancy. Because of small numbers, we did not examine the outcomes of previous pregnancies in these data. However, Cameron County women may have had more pregnancies that did not result in a live birth. Half of all abortions in the United States occur among women younger than 25 years, and abortion rates among Hispanic women in the United States are increasing (15). Women in Matamoros may have been less likely than those in Cameron County to report a previous pregnancy that did not result in a live birth, since most abortions are illegal in Mexico (16-18).

The large proportion of unintended and repeat pregnancies are cause for concern. The situation appears to be somewhat worse in Cameron County, where more of the women were single and lacked health insurance at the time of conception. Possible contributors to the problem in Cameron County include higher rates of alcohol consumption (19), lower crude rates of postpartum contraception counseling during prenatal care, and lower rates of breastfeeding, which reduces fertility temporarily. Additional analysis of survey data revealed that multigravida women in Cameron County reported a median interval of 24 months between the current live birth and the birth of the previous child, whereas the median interval for Matamoros women was 36 months.

The overall high proportion of unintended pregnancies is related to the low rates of contraception use both at first sexual intercourse (34.0%) and at conception (40.7%). Unintended pregnancy in this population may also be related to ineffective use of contraception, given that so many women reported use of contraception at conception. By comparison, US Hispanic females aged 15 to 19 years are nearly twice as likely to have used contraception at first sexual intercourse (66%) (20), and similarly large proportions of female US Hispanic (55.5%) and Tamaulipas (54.3%) adolescents report using a condom at first sexual intercourse (20,21).

Condoms were the most common first method of contraception among both Matamoros and Cameron County women. The higher prevalence of IUD use in Matamoros may be due to greater emphasis on IUDs in public family planning services throughout Mexico, including Tamaulipas (18). Use of a method such as an IUD that is provider-administered and requires planning may also be more common among women who are married or cohabiting with their partners and among women with health insurance, who represented a larger proportion of the Matamoros than the Cameron County sample. While the IUD and injectable hormones offer greater long-term protection against pregnancy, neither protects against STIs, and rates of STIs are thought to be high and increasing in the border region (22-24). Although almost all study women knew at least 1 way to prevent HIV infection, almost 40% used a nonbarrier method of contraception. This survey did not include questions about dual use of contraceptive methods.

The rates of smoking at the time of pregnancy among the women in this study, most of whom were born in Mexico, were considerably lower than those reported by all US (25) and Texas (26) Hispanic childbearing women (10.5% and 8.4%, respectively) in the Pregnancy Risk Assessment Monitoring System (PRAMS). Similarly, in preliminary Texas PRAMS data from 2005, 8.5% of Hispanic women aged 14 to 24 years reported smoking 3 months before giving birth (Eric Miller, PhD, MSPH, written communication, March 2008). These rates differ dramatically from current smoking rates reported by US Hispanic female high school students (19.2%) (19). In Tamaulipas, we did not measure current smoking, but 8.6% of all adolescent women reported having ever smoked (21). The higher rates of smoking among Hispanic women in Texas and the United States were lower than rates for US women overall (19,25-26) and may have resulted from increased levels of acculturation and years of residence in the United States (27).

The prevalence of alcohol use in Cameron County (38.4%) was comparable to that of young Hispanic women in Texas PRAMS data (35.8%) (26) and Hispanic female high school students in the United States (44.8%) (19). Current alcohol use among Matamoros women in this survey (15.3%) was lower than lifetime prevalence of alcohol use among females aged 10 to 19 years in Tamaulipas (27.3%) (21). Cameron County women were more likely to have used alcohol than were Matamoros women despite the fact that the legal drinking age in Mexico is 18, compared with 21 in the United States. This difference could also be due to acculturation of young Cameron County women, or greater reluctance to admit drinking by women in Mexican society. Drinking was more common among Cameron County women who spoke English (46%) than those who spoke Spanish (29%) (data not shown).

Cultural factors, such as a less favorable attitude toward breastfeeding in the United States, may contribute to the lower prevalence of breastfeeding in Cameron County than in Matamoros (28). This difference may also result from the lack of any national policy on breastfeeding in the United States, in contrast to very strong policies supporting breastfeeding in Mexico (29), and the provision of discounted infant formula to women in Cameron County hospitals (28). The breastfeeding prevalence in Matamoros was comparable to the prevalence reported by Tamaulipas adolescents in the mid-1990s (78.1%) (18). The 62.6% weighted prevalence of hospital breastfeeding among Cameron County women aged 14 to 24 years is consistent with findings from another study conducted in Texas in 2007 that showed a prevalence of 61.2% in this age group (30). Increased and improved educational interventions to promote breastfeeding are needed in the United States.

Most young women on both sides of the border had health care coverage during pregnancy and received prenatal care. Rates of early prenatal care in Cameron County were lower than those for all US women and nearly identical to rates among US Hispanic women (25). The 57.9% prevalence of first trimester prenatal care in Matamoros was lower than the 73.0% prevalence reported for all Tamaulipas adolescents in an earlier survey (18). The low prevalence of cervical cancer screening in Matamoros may be because such screening is not a routine part of prenatal care for young women in Mexico (31). The most remarkable feature of the clinical care received by these women is the high prevalence of cesarean births in both communities. These levels are higher than overall US and Tamaulipas rates (18,32) and much higher than what is considered optimal (15%) (33).

Despite the high rates of cesarean births, the gaps in prenatal care in both communities, and the low educational attainment, there is little indication in these data that the prevalence of low birth weight or preterm birth in these communities is substantially different from that of the Mexican or US population as a whole (34,35). This phenomenon has been termed the "Hispanic paradox" (36) and has been noted among US Hispanics even after adjustment for the lower prevalence of smoking among Hispanic women.

The overall strengths and limitations of the BMSCP that are discussed elsewhere (10) apply to this analysis. A contribution of this particular analysis is the way it helps isolate the effects of the social and health care environment on the pregnancies of groups of adolescents and other young women who have common genetic and cultural traits. The study's major weakness is the small number of adolescents, which limited the possibilities for special analysis of this age group. A second weakness is potential response bias from social pressure to avoid revealing undesirable behavior, especially with the stigma already associated with pregnancy among adolescents and single women.

Renewed efforts are needed to reduce the rates of unplanned pregnancies among adolescents living in the US-Mexico border region, perhaps through the creation of programs to increase the use of contraception. Both communities need to provide low-cost health care coverage both before and during pregnancy. Increasing the percentage of women enrolled in prenatal care early should remain a priority. Preconception and postconception reproductive health care should incorporate information about high-risk behaviors such as smoking and alcohol consumption and increase rates of cervical cancer screening. Hospitals should encourage breastfeeding and reduce the rates of cesarean births. Several concerns identified in this analysis are evident on both sides of the border, and many of the young mothers and fathers involved have occupational, social, and familial ties in both countries; a joint effort of sister cities to address these concerns, employing a consistent binational and bilingual approach, would have many advantages.

## Acknowledgments

The BMSCP was funded through CDC's Division of Reproductive Health and the Office of Global Health Promotion at the National Center for Chronic Disease Prevention and Health Promotion, under a cooperative agreement with the United States-Mexico Border Health Association, No. U65 CCU 623699-01-2, and through interagency personnel agreements with the University of Texas at Brownsville and Texas Southmost College and the University of Texas-Houston School of Public Health, Brownsville Regional Campus. In-kind project support was provided by CDC's Division of Health Examination Statistics at the National Center for Health Statistics; the Texas Department of State Health Services, Region 11; the Secretariat of Health, Tamaulipas; and the Mexican Institute of Social Security, Tamaulipas.

Support from the following local, regional, and national institutions was critical to the project: the National Center for Gender Equity and Reproductive Health, Mexican Health Secretariat; National Center for Epidemiologic Surveillance and Disease Control, Mexican Health Secretariat; National Center for Health Promotion, Mexican Health Secretariat; National Institute of Statistics, Geography and Informatics, Tamaulipas; Civil Registry, Tamaulipas; Institute for Social Security and Services for State Workers, Tamaulipas; Secretariat of Health, Jurisdiction III, Tamaulipas; Texas Department of State Health Services, Region 11 and Office of Border Health; City of Brownsville Department of Public Health; Cameron County Health Department; Valley Baptist Medical Center in Harlingen; Valley Baptist Medical Center in Brownsville; Valley Regional Medical Center; Harlingen Medical Center; Cameron Park Cultural Center; Brownsville Community Health Center; Dr. Alfredo Pumarejo Lafaurie, General Hospital of Matamoros; Mexican Institute of Social Security General Hospital, Zone #13, Matamoros; Dr Manuel F. Rodríguez Brayda Clinical Hospital, Matamoros; Hospital Guadalupe; Matamoros Center of Family Orientation; Medical Center of Surgical Specialties of Matamoros, and the United States-Mexico Border Health Commission.

We thank Dr Ruben Smith of the Division of Reproductive Health, CDC, for statistical assistance with birth rate calculations; Dr Eric Miller, PRAMS Coordinator at the Texas Department of State Health Services, for providing PRAMS data and comments on the manuscript; and Dr Ushma Upadhyay for her review of the manuscript. Special thanks to the National Center for Gender Equity and Reproductive Health, Secretariat of Health, Mexico, for coordinating review of this manuscript in Mexico and to the United States-Mexico Border Health Commission for providing the English-Spanish translation.

## Figures and Tables

**Table 1:** Comparison of Age-Specific Birth Rates Among Women Aged 14-24 Years in Matamoros, Tamaulipas, Mexico, and Cameron County, Texas, 2005^a^

Age, y	No. of BMSCP Survey Births^b^	Estimated No. of Population Births, Weighted (95% CI)^c^	No. of Births/1,000^d^(95% CI)	No. of Births/1,000 From Vital Statistics^e^
**Matamoros**				
10-19^f^	94	2,395 (1956-2834)	54.3 (44.4-64.3)	37.4
15-19	91	2,318 (1843-2794)	110.6 (88.0-133.3)	74.9
20-24	154	3,926 (3208-4641)	190.2 (155.4-224.8)	125.9
Total	248	6,322 (5407-7232)	97.7 (83.5-111.7)	67.2
**Cameron County**				
10-19	67	1,539 (1167-1911)	47.9 (36.3-59.4)	50.6
15-19	66	1,517 (1163-1870)	97.5 (74.7-120.2)	102.6
20-24	141	3,244 (2681-3808)	213.1 (176.1-250.1)	179.2
**Total**	208	4,781 (4092-5475)	100.9 (86.4-115.6)	90.4

Abbreviations: BMSCP, Brownsville-Matamoros Sister City Project for Women's Health; CI, confidence interval.

a Age-specific birth rate estimates are calculated by dividing estimates of the number of live births in an age-defined population in a year by estimates of the midyear resident population in the defined age group.

b The actual number of live births that occurred during the 81-day study period to women in the study sample.

c An estimate of the number of live births that occurred to women in each age group during 2005 calculated from survey data. Estimates of the number of live births that occurred to women in each age group were weighted to approximate the population of women who had a live birth during the 81-day study period in Matamoros and in Cameron County and corresponding 95% CIs were calculated. Estimates and corresponding 95% CIs were then annualized by multiplying the weighted population estimate and associated standard errors by 4.51 (365 days/81 study period days).

d Birth rate estimates from survey data were calculated with the 2005 estimate of number of live births from the previous column as the numerator and age-specific midyear population estimates for women as the denominator. Midyear population estimates for denominator data for Cameron County were obtained from the US Census Bureau (12) and for Matamoros, from the National Institute of Statistics, Geography and Informatics (11).

e Age-specific birth rates estimated from vital statistic data use the total annual number of births reported as the numerator and age-specific midyear population estimates for women as the denominator. For Cameron County, birth rates were calculated based on preliminary 2005 vital statistics numerator data (J. Jackson, MPH, written communication, February 2008) and 2005 census denominator data (12); for Matamoros, 2006 vital statistics numerator data (6) and 2006 census denominator data (7) were used because 2005 vital statistics were not available.

f The number of women aged younger than 15 years was too small for separate analysis.

**Table 2:** Sociodemographic Characteristics of Women Aged 14-24 Years Who Gave Birth in the US-Mexico Border Region, Brownsville-Matamoros Sister City Project for Women's Health, 2005^a^

Characteristic	Place of Residence		Total (N = 456)^b^ No. (%)	*P* value^c^

	Matamoros (n = 248) No. (%)	Cameron County (n = 208) No. (%)		
**Age, y (n = 456)**				.19
14-19	531 (37.9)	342 (32.2)	873 (35.4)	
20-24	871 (62.1)	720 (67.8)	1,591 (64.6)	
**Ethnicity (n = 451)**				<.001
Not Hispanic	0 (0)	139 (13.4)	139 (5.7)	
Hispanic	1,403 (100.0)	897 (86.6)	2,300 (94.3)	
**Place of birth (n = 452)**				<.001
Mexico, Tamaulipas	893 (64.1)	302 (28.7)	1,195 (48.9)	
Mexico, Other	493 (35.4)	123 (11.7)	616 (25.2)	
United States, Texas	0	545 (51.9)	545 (22.3)	
United States, Other	6 (0.4)	81 (7.7)	87 (3.5)	
**Interview language (n = 456)**				<.001
Spanish used	1,397 (99.6)	482 (45.4)	1,879 (76.2)	
No Spanish used	6 (0.4)	580 (56.4)	585 (23.8)	
**Marital status (n = 453)**				<.001
Single	169 (12.1)	352 (33.3)	521 (21.3)	
Married/living together	1,223 (87.9)	704 (66.7)	1,927 (78.7)	
**Education (n = 426)**				<.001
<8th grade	415 (31.6)	56 (5.6)	470 (20.4)	
8th-12th grade	786 (59.9)	491 (42.5)	1,277 (55.5)	
High school graduate	112 (8.5)	443 (44.8)	555 (24.1)	
**Employment status (n = 453)**				.27
Unemployed	85 (6.1)	113 (10.8)	198 (8.1)	
Employed	630 (44.9)	445 (42.5)	1,075 (43.9)	
Not in labor market	688 (49.0)	489 (46.7)	1,177 (48.0)	
**Health care coverage before pregnancy (n = 456)**				<.001
Yes	699 (49.8)	193 (18.1)	891 (36.2)	
No	704 (50.2)	869 (81.9)	1,573 (63.8)	
**Health care coverage during pregnancy (n = 456)**				.33
Yes	898 (64.0)	724 (68.2)	1,622 (65.8)	
No	504 (36.0)	338 (31.8)	842 (34.2)	

a Numbers are weighted population counts and therefore are greater than the total sample size of the survey. Percentages take population weights into account.

b Survey sample sizes are <456 for some variables because of missing data.

c Calculated by using the Pearson χ^2^ method.

**Table 3:** Reproductive Health Characteristics of Women Aged 14-24 Years Who Gave Birth in the US-Mexico Border Region, Brownsville-Matamoros Sister City Project for Women's Health, 2005^a^

Characteristic	Place of residence		Total (N = 456)^b^ No. (%)	OR (95% CI)	AOR^c^ (95% CI)

	Matamoros, Mexico (n = 248) No. (%)	Cameron County, United States (n = 208) No. (%)			
**Gravidity (n = 456)**					
1st pregnancy	774 (55.2)	480 (45.2)	1,254 (50.9)	1.00	1.00
≥1 prior pregnancies	629 (44.8)	582 (54.8)	1,210 (49.1)	0.67 (0.46-0.97)	0.27 (0.15-0.47)
**Pregnancy intention (n = 448)**					
Intended	662 (47.4)	377 (36.8)	1,040 (42.9)	1.00	1.00
Unintended	735 (52.6)	648 (63.2)	1,383 (57.1)	0.65 (0.45-0.92)	0.94 (0.58-1.54)
**Low birth weight (<2,500 g)(n = 454)**					
No	1,295 (93.1)	969 (91.3)	2,265 (92.3)	1.00	1.00
Yes	96 (6.9)	92 (8.7)	188 (7.7)	0.78 (0.4-1.5)	0.64 (0.23-1.77)
**Preterm birth (<37 weeks' gestation)(n = 455)**					
No	1,106 (93.3)	893 (85.4)	1999 (89.6)	1.00	1.00
Yes	80 (6.7)	153 (14.6)	233 (10.4)	0.42 (0.17-1.05)	0.49 (0.15-1.58)
**Method of delivery (n = 455)**					
Vaginal	849 (60.8)	664 (62.5)	1,513 (61.5)	1.00	1.00
Cesarean	548 (39.2)	398 (37.5)	946 (38.5)	1.08 (0.75-1.54)	1.38 (0.85-2.22)
**Breastfeeding initiated (n = 456)**					
No	227 (16.2)	397 (37.4)	623 (25.3)	1.00	1.00
Yes	1,176 (83.8)	665 (62.6)	1,841 (74.7)	3.01 (1.89-5.09)	4.00 (1.92-8.35)
**Prenatal care (n = 448)**					
None/2nd or 3rd trimester	576 (42.1)	317 (30.1)	893 (36.9)	1.00	1.00
1st trimester	792 (57.9)	735 (69.9)	1,527 (63.1)	0.59 (0.42-0.83)	0.50 (0.30-0.85)
**Postpartum contraception counseling during prenatal care^d^ (n = 435)**					
No	400 (30.6)	430 (41.2)	830 (35.4)	1.00	1.00
Yes	906 (69.4)	612 (58.8)	1,518 (64.6)	1.59 (1.01-2.39)	1.16 (0.69-1.93)
**Contraception use at conception^e^ (n = 266)**					
No	417 (58.5)	430 (60.1)	847 (59.3)	1.00	1.00
Yes	295 (41.5)	286 (39.9)	581 (40.7)	1.07 (0.67-1.71)	1.28 (0.61-2.70)
**Contraception at first sexual intercourse (n = 447)**					
No	1,030 (74.3)	567 (55.0)	1,597 (66.0)	1.00	1.00
Yes	357 (25.7)	464 (45.0)	821(34.0)	0.42 (0.28-0.65)	0.68 (0.37-1.27)
**Contraceptive method first used^f^ (n = 297)**					
Barrier (condom/ diaphragm)	453 (57.6)	505 (62.7)	958 (60.2)	1.00	^1.00^
Pill or patch	113 (14.4)	183 (22.8)	296 (18.6)	0.69 (0.34-1.39)	ND^g^
Injection	61 (7.8)	92 (11.4)	153 (9.6)	0.75 (0.34-1.64)	ND^g^
Intrauterine device	137 (17.4)	25 (3.1)	162 (10.2)	6.02 (1.94-18.7)	ND^g^
Withdrawal or rhythm	23 (2.9)	0 (0.00)	23 (1.4)	ND^g^	ND^g^
**Knowledge of HIV prevention methods (n = 399)^h^**					
Neither method	40 (3.1)	31 (3.5)	71 (3.3)	1.00	1.00
1 method	961 (75.1)	751 (85.1)	1,712 (79.2)	0.98 (0.36-2.72)	ND^g^
Both methods	278 (21.8)	101 (11.5)	380 (17.6)	2.11 (0.60-7.38)	ND^g^
**High-risk behavior (n = 454)**					
No	1,324 (94.4)	984 (93.6)	2,308 (94.0)	1.00	1.00
Yes	79 (5.6)	67 (6.4)	146 (6.0)	0.88 (0.43-1.80)	0.86 (0.38-1.94)
**Smoking 3 months before pregnancy (n = 428)**					
No	1,329 (99.6)	969 (98.9)	2,299 (99.3)	1.00	1.00
Yes	6 (0.4)	10 (1.1)	16 (0.7)	0.40 (0.03-4.58)	3.17 (0.54-18.6)
**Alcohol use 3 months before pregnancy (n = 453)**					
No	1,183 (84.7)	648 (61.6)	1,831 (74.8)	1.00	1.00
Yes	214 (15.3)	403 (38.4)	617 (25.2)	0.29 (0.20-0.43)	0.52 (0.23-0.93)
**Ever had a Pap test (n = 454)**					
No	782 (55.8)	73 (7.0)	856 (34.9)	1.00	1.00
Yes	620 (44.2)	978 (93.0)	1,598 (65.1)	0.06 (0.04-0.10)	0.03 (0.01-0.08)

Abbreviations: OR, odds ratio; AOR, adjusted odds ratio; CI, confidence interval; ND, not determined; HIV, human immunodeficiency virus; Pap, Papanicolaou.

a Numbers are weighted population counts and therefore are greater than the total sample size of the survey. Percentages take population weights into account.

b Total sample sizes are <456 for some variables because of missing data.

c Adjusted for ethnicity, age (modeled as a continuous variable), education, marital status, and health care coverage before pregnancy.

d Of the 436 women who responded that they had received some prenatal care, 435 responded to the question regarding whether a doctor, nurse, or other health care worker talked about birth control methods to use after pregnancy during any prenatal care visits.

e Of the 267 women who did not respond that they were trying to get pregnant, 266 responded to the question regarding the use of any contraception at the time of conception.

f Of the 307 women who did not report that they never used any birth control, 297 provided information regarding the contraceptive method first used.

g Values were too small to compute AOR.

h The 2 methods were defined as 1) using a condom and 2) limiting sex/staying faithful to 1 partner.
